# Chitosan in Oral Drug Delivery Formulations: A Review

**DOI:** 10.3390/pharmaceutics15092361

**Published:** 2023-09-21

**Authors:** Tanikan Sangnim, Divya Dheer, Nitin Jangra, Kampanart Huanbutta, Vivek Puri, Ameya Sharma

**Affiliations:** 1 Faculty of Pharmaceutical Sciences, Burapha University, Chonburi 20131, Thailand; tanikan@go.buu.ac.th; 2 Chitkara School of Pharmacy, Chitkara University, Baddi 174103, Himachal Pradesh, India; divya.dheer@chitkarauniversity.edu.in (D.D.);; 3 Chemical Biology Unit, Institute of Nano Science and Technology, Knowledge City, Sector 81, Mohali 140306, Punjab, India; 4 Department of Manufacturing Pharmacy, College of Pharmacy, Rangsit University, Pathum Thani 12000, Thailand

**Keywords:** chitosan, nanoformulations, oral drug delivery, herbal bioactives, patents

## Abstract

Nanoformulations have become increasingly useful as drug delivery technologies in recent decades. As therapeutics, oral administration is the most common delivery method, although it is not always the most effective route because of challenges with swallowing, gastrointestinal discomfort, low solubility, and poor absorption. One of the most significant barriers that medications must overcome to exert a therapeutic effect is the impact of the first hepatic transit. Studies have shown that controlled-release systems using nanoparticles composed of biodegradable natural polymers significantly improve oral administration, which is why these materials have attracted significant attention. Chitosan possesses a wide variety of properties and functions in the pharmaceutical as well as healthcare industries. Drug encapsulation and transport within the body are two of its most important features. Moreover, chitosan can enhance drug efficacy by facilitating drug interaction with target cells. Based on its physicochemical properties, chitosan can potentially be synthesized into nanoparticles, and this review summarizes recent advances and applications of orally delivered chitosan nanoparticle interventions.

## 1. Introduction

**Figure 1:**
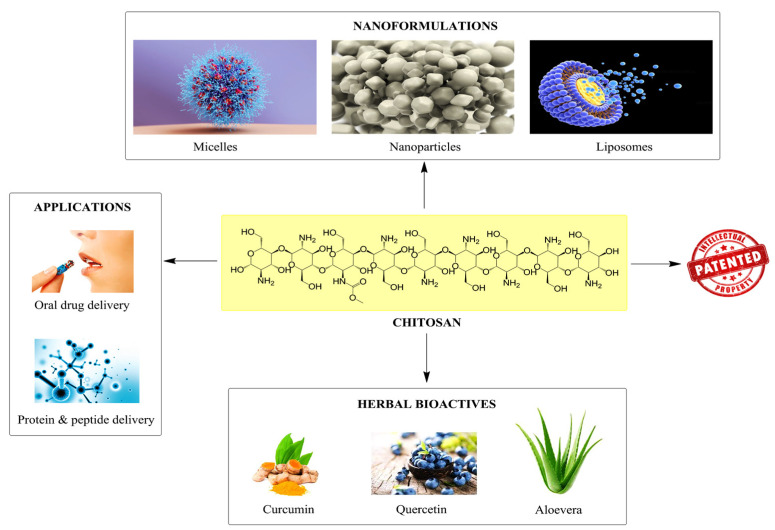
Use of herbal bioactives in chitosan-infused nanoformulations has resulted in novel applications and patented innovations.

In recent decades, there has been increased interest in developing innovative methods to deliver drugs. In this review, we will discuss the pros and cons of drug delivery systems, the basic as well as physicochemical characteristics that could render a drug suitable for pharmaceutical formulation, and the techniques used to assess delivery sustainability, toxicity at the site of delivery, and its feasibility [1]. Despite the abundance of research on various methods for administering drugs, oral administration is still the most efficient, least complicated, and safest [2,3]. The advent of nanotechnology, and particularly, the field of nanomedicine, has increased the acceptance of nanocarriers as oral drug delivery vehicles, thereby overcoming these restrictions. The utilization of nanometric particles is at the core of nanomedicine, which is considered a branch of nanotechnology [4]. Numerous types of drug delivery systems make use of nanoparticles (NPs) as drug carriers. The pharmaceutical industry has found NPs to be useful for treating diseases such as cancer, diabetes, and HIV [5,6]. In order to improve therapeutic constraints and membrane crossing, NPs were developed. There is concern that some of the synthetic compounds used in these products present health risks to individuals [7]. Nanoparticles developed from polymers, such as chitosan NPs, and the infusion of herbal bioactives (Curcumin, aloe vera, etc.) possess desirable properties and applications because of their inherent versatility, biological compatibility, and biodegradability (Figure 1) [8,9].

There are a wide variety of polymers, both natural, synthetic, and semisynthetic, that can be used to formulate nanoparticles for various drug delivery [10]. Chitosan and its derivatives are distinguished from other polymers because of their superior characteristics, making them ideal for oral administration [11]. The addition of amino groups imparts a cationic charge, which enhances penetration and acts as a mucoadhesive [12]. These NPs are also non-toxic, biocompatible, and biodegradable. The biopolymer chitosan (CS) has gained significant attention in the medical field because of its versatility, availability, and unique properties. The linear polysaccharide chitosan is comprised of N-acetyl-D-glucosamine and D-glucosamine units that are randomly repeated and linked together via (1→4) glycosidic linkages [13]. Chitin is the source of chitosan, a natural polymer that is second only to cellulose in natural abundance. Chitin and chitosan are widely used in various industries, including the food industry as well as medical and pharmaceutical fields (i.e., tissue engineering, gene transplantation, and wound healing) [14]. Chitosan nanoparticles (CSNPs) have intriguing properties, particularly for ocular and oral distribution. Some active components are not very stable or bioavailable; however, CSNPs are considered a promising vehicle for improving these factors. To enhance retention and cellular uptake, chitosan can adhere strongly to the negatively charged mucus membrane [15]. Interestingly, many research studies were published related to chitosan drug delivery in the last few years. Recently, Guadarrama-Wacobar et al. 2023, reported chitosan-based oral delivery, mostly covering 2020 research data, and we focused on most of the research data that includes references from 2022 onwards. Now, we have reorganized our manuscript with the following subheadings, namely Introduction, Oral drug delivery, Nanotechnology in delivery systems, Applicability of chitosan-oriented multifarious delivery, Role of P-gp inhibitors in oral drug delivery, herbal bioactive-loaded nanoformulations in oral drug delivery, Patents, Future perspectives, and Conclusion.

The manuscript is structured based on the recent advancements targeting different chitosan-based oral nanodrug delivery systems in a concise way that covers most of the relevant areas.

## 2. Oral Drug Delivery

Prior to the dosage, it is important to think about how the drug will interact with the patient’s body [16]. The drug’s characteristics and how well it is absorbed will determine the best method of administration for maximizing bioavailability and efficacy. Medical professionals are generally in charge of administering drugs, as opposed to writing prescriptions [17]. Drug delivery systems provide properties such as targeted distribution and the controlled release of therapeutic drugs [18]. Drug delivery systems, such as nanocarriers, are typically composed of lipids or polymers as well as associated therapies and can improve many of the pharmacologic properties of free medicines [19]. Some drug delivery systems are intended to act as reservoirs (i.e., sustained-release systems), whereas others are designed to modify the pharmacokinetics and biodistribution of the compounds they deliver [20].

The therapeutic application of numerous types of nanoparticles is a promising new direction in drug delivery and nanotechnology, which is at the forefront of this field [21]. The United States Environmental Protection Agency defines nanotechnology as “the development and implementation of structures, devices (medicated or implants), as well as delivery systems, because of their small size, having distinctive novel features as well as functions” [22].

The digestive tract consists of a number of different administration sites for oral medications [23]. The inherent characteristics of the gastrointestinal route allow for a wide range of habitats, each with its own set of advantages and disadvantages. The fate of a drug is highly dependent on factors, such as its pH, the availability of absorption sites, the presence of digestive enzymes, and the amount of free water [24]. One of the obstacles to overcome is the varying oral absorption site. The pH of the gastrointestinal (GI) tract plays a crucial role in oral drug delivery and the absorption of medications. The GI tract consists of various regions, each with a distinct pH environment [25].

Starting from the oral cavity, the pH is generally around 6 to 7, slightly acidic to neutral. As the drug progresses through the stomach, the pH drops drastically due to the presence of gastric acid, reaching a highly acidic pH of 1 to 3. This low pH can affect the stability of certain drugs, and formulations with enteric coatings are often used to protect them from degradation in the stomach. Moving further down to the small intestine, the pH gradually increases to a more alkaline range of 6 to 7.5. This region is the primary site for drug absorption due to its large surface area and extensive blood supply. The pH of the large intestine ranges from 5.5 to 7, slightly acidic to neutral. Understanding the pH variations in different GI tract regions is essential for formulating oral drug delivery systems [26,27]. It enables researchers to design drug formulations that can withstand the acidic environment of the stomach and optimize drug release and absorption in the intestines. There are more challenges to contemplate, such as [28,29,30,31,32], which are as follows:

- The risk of the administered medicine becoming unstable is increased by the presence of digestive enzymes in the gastrointestinal tract. In the context of using large molecules, such as proteins, and nucleic acids, this is a particularly significant concern.
- The probability of absorption of macromolecules by gastrointestinal cells is low.
- Food intake may alter drug absorption because the duration of stomach emptying is proportional to the amount of food consumed.
- There is often no precise delivery location, making it difficult to exercise control over the drug release.
- The parenteral or rectal route of drug delivery is still the preferred route for some patients, although oral administration is the most common.

As a result, the properties of stability against degradation, regulated drug release profile, and targeted site of absorption/action are important features of an effective oral delivery system [33].

## 3. Nanotechnology in Delivery Systems

The polymer aids in wound healing by enhancing hemostasis and accelerating tissue regeneration and it is degraded by human enzymes. It has been used in various biological fields, such as wound healing systems, oral delivery systems, and orthopedics. It may be fused with various polymeric biomaterials along with inorganic bioactive compounds for the development of bone graft substitutions, intervertebral disks, cartilage, and bone-related tissue engineering materials [34,35,36].

### 3.1. Nanoparticles

Nanomaterials such as nanoparticle-loaded oral films in combination with chitosan/sodium alginate and curcumin (CUR) can be employed to treat oral biofilms with antimicrobial photodynamic therapy (aPDT). Silvestre and his team in 2023 developed and evaluated a novel nanoparticle delivery system for the treatment of aPDT, consisting of CUR-encapsulated chitosan and sodium alginate. The NPs were synthesized using polyelectrolytic complexation, while the biofilm formed via solvent evaporation. The efficiency of the photodynamic effect was measured in terms of colony-forming units per milliliter (CFU/mL). When comparing nanoparticle-loaded films to nanoparticles in an artificial saliva medium (in vitro), the latter approach showed superior control over CUR release. These results lend credence to the idea that aPDT-coupled chitosan/sodium alginate nanoparticles could be useful for the oral delivery of CUR. This paves the way for the development of cutting-edge oral delivery techniques, and it could one day be used to treat things such as dental cavities and infections [37]. Fan et al. [38] developed mucoadhesive chitosan-based nanoparticles for the oral delivery of low molecular weight heparin with enhanced bioavailability and anticoagulant effects for the prevention of deep venous thrombosis. However, there are a number of limitations that make such treatments difficult to administer orally. To prevent low molecular weight heparin (LMWH) breakdown and improve its absorption via the gastrointestinal mucosa, a pH-responsive delivery system was developed. Ionic crosslinking between the positively charged amino groups of thiolated chitosan (TCS), *O*-carboxymethyl chitosan (O-CMCS), and negatively charged HP55 was carried out to generate LMWH-loaded nanoparticles (pH-TCS/O-CMCS@LMWH). With an average size of 332 nm, a high encapsulation effectiveness of 96.6%, and a drug-loading concentration of 12.04 IU/mg, the resulting nanoparticles were found to adopt a spherical form. The suggested pH-TCS/O-CMCS@LMWH exhibited favorable pH-responsive drug release behavior, which reduced premature LMWH release into the acidic environment, while increasing its targeted release in the intestinal environment, in addition to its high colloidal stability and possible mucoadhesive action. Increased bioavailability was achieved and considerable antithrombotic activity of pH-TCS/O-CMCS@LMWH following oral administration in a rat model of venous thrombosis was observed. Thus, pH-TCS/O-CMCS nanoparticles show significant promise as a means of oral delivery of LMWH for the treatment of thrombosis.

Nanotechnology plays an integral role in multimodal analgesia. In this direction Wasana and his team in 2023 co-encapsulated metformin (Met) and curcumin (Cur) into chitosan/alginate (CTS/ALG) nanoparticles (NPs) at their synergistic drug ratio by applying response surface methodology. The synthesized Met-Cur-CTS/ALG-NPs had a Met/Cur mass ratio of 2.9:1, a particle size of 243 nm, a zeta potential of −21.6 mV, an encapsulation efficiency of 32.6% and 44.2%, and drug-loading Met of 6.8% and Cur of 19.6%, respectively. Met-Cur-CTS/ALG-NPs were stable in simulated GI fluid after being stored for an extended period of time. Met-Cur-CTS/ALG-NPs showed sustained release in an in vitro study of simulated gastrointestinal fluids, with Met exhibiting Fickian diffusion and Cur exhibiting non-Fickian diffusion according to the Korsmeyer-Peppas model. The mucoadhesion and cellular absorption of Met-Cur-CTS/ALG-NPs in Caco-2 cells were significantly enhanced. Met-Cur-CTS/ALG-NPs were also found to have a greater capacity to modulate peripheral and central immune mechanisms of pain, as evidenced by their superior anti-inflammatory effects in lipopolysaccharide-stimulated RAW 264.7 macrophage and BV-2 microglial cells. Met-Cur-CTS/ALG-NPs suppressed pain-like responses and proinflammatory cytokine production more effectively than the Met-Cur physical combination in a mouse model of formalin-induced pain. In addition, therapeutic doses of Met-Cur-CTS/ALG-NPs were not associated with any serious adverse effects in mice. In conclusion, the current study establishes a CTS/ALG nano-delivery method for the Met-Cur combination for enhanced efficacy and safety in the treatment of pain [39].

Enoxaparin is a potent biological compound for the treatment and prevention of bleeding; however, its absorption in the gut is relatively low. Without using high-energy homogenization or any organic solvents, Patriota and co-workers synthesized Eudragit^®^ L100 (Evonik Rohm GmbH, Darmstadt, Germany) coated chitosan core-shell nanoparticles for enoxaparin oral administration. The particles were less than 300 nm in size, exhibited a polydispersity index of about 0.12, a zeta potential of more than +25 mV, an entrapment efficiency greater than 95%, and negligible cumulative enoxaparin release (10%) when subjected to simulated gastric fluid conditions, thus demonstrating the success of the Eudragit^®^ L100-coating process. These results indicate that the enteric-coating method and drug delivery nanotechnology are viable options for enoxaparin oral administration [40]. Nanoparticles have gained prominence as a promising drug delivery technology in recent years. Oral administration is the most common method for therapeutic treatments, despite the fact that it is not always the most effective route due to issues such as difficulty swallowing, gastrointestinal discomfort, low solubility, and poor absorption. One of the most significant barriers that drugs have to overcome to have their therapeutic effect is the impact of the first hepatic pass. As a result, many studies have found that controlled-release systems based on nanoparticles synthesized from biodegradable natural polymers significantly improve oral delivery. One of the most important functions of chitosan in the pharmaceutical and health fields is its ability to encapsulate and transport drugs within the body and to improve the drug’s interaction with the target cells, thus increasing the drug’s efficacy [41].

### 3.2. Liposomes

To transport both small molecules and macromolecules, liposomes may be used as a drug carrier. Phosphatidylcholine and cholesterol are frequently used as liposome constituents. They have the capacity to store chemicals that can be maintained in either water or fat. Liposomes have a hydrophilic layer or inner cavity, where soluble molecules are retained, and a lipid wall, where low water- or oil-soluble compounds may be stored. Liposomes have the potential to enable the controlled and prolonged release of drugs, which can boost a drug’s efficacy and therapeutic index. There are potential benefits to loading protein drugs into liposome formulations, including improved stability, prolonged release, less degradation, and increased penetration [42]. Phospholipids and cholesterol are the building blocks of liposomes, which are tiny spherical vesicles. Several liposomal formulations have exhibited clinical activity; however, liposomes are rarely used for the oral administration of drugs, in part, because traditional liposomes are susceptible to gastrointestinal destabilizing agents and inefficient intestinal absorption. Layer-by-layer assembly technology, which has been widely used to modify the surface of various nanoparticulate systems, may overcome some of these problems [43].

In 2023, Wang and colleagues optimized the use of electrostatic deposition for developing pectin and chitosan double-layer-coated liposomes to increase storage stability and gastrointestinal (GI) stability. The results indicated that pectin and chitosan double layer-coated liposomes may be prepared with 0.2% chitosan and 0.06% pectin with good outcomes. After electrostatic connection, the structure of pectin and chitosan double-layer-coated liposomes was maintained with hydrogen bonds between the amino groups in chitosan and the liposomal interfacial area, and by interactions between the carboxyl groups in the pectin layer and the amino groups in the chitosan layer. The chemical stability of the encapsulated β-carotene (βC) and liposome thermal stability may be enhanced with double-layer coatings. Furthermore, the polymer coating altered the permeability of the liposomal bilayer and the mechanism of βC release in simulated GI fluids. Compared with chitosan double-layer-coated liposomes and liposomes, pectin and chitosan double-layer-coated liposomes exhibited more precise control over the release of βC, and had a positive effect on the delivery of bioactive compounds via the intestinal tract. This could assist in the development of more effective bioactive agent delivery systems [44].

Preventing drug release or breakdown in the stomach or small intestine and transporting it to the colon, where colon cancer is most prevalent, are two major challenges associated with 5-fluorouracil (5-FU) administration. Targeted treatment of colon cancer may be possible with the development of liposomes utilizing a suitable ligand and encapsulation. Khodarahmi et al. [45] used an anti-nucleolin aptamer (AS1411) as a ligand to synthesize targeted liposomes by a thin film approach. The resulting liposomes were then coated with an alginate and chitosan solution to produce nanocapsules. The cytotoxicity of HT-29 colon cancer cells was assessed in vitro using an MTT assay. Both the liposomes and nanocapsules were spherical and 120–170 nm in size. Cell death by aptamer-conjugated liposomes occurred at much higher rates compared with aptamer-free liposomes or free drugs.

Chitosan was inserted into the liposome core to increase the effectiveness of niacin loading and enhance the stability of lecithin. Niacin release can be further suppressed at physiological pH 7.4 using the hydrophobic chemical curcumin as a face layer encased in a liposomal vesicle. Liposomes were delivered to cancer cells more precisely using chitosan coupled with folic acid. After a 48 h incubation at 100 μg/mL, the growth rate of HePG2 was significantly inhibited by 91 ± 1% (pure niacin), 55 ± 3% (pure curcumin), 83 ± 1.5% (niacin NPs), and 51 ± 1.5% (curcumin-niacin NPs) compared with the control (based on cellular proliferation). A significant fold-increase in the expression of mTOR mRNA was observed (0.72 ± 0.08 *p* ≤ 0.001, 1 ± 0.10, *p* ≤ 0.001, 5 ± 0.07 *p* ≤ 0.01, and 1.3 ± 0.02 *p* ≤ 0.001) following treatment with pure niacin, pure curcumin, niacin NPs, and curcumin-niacin NPs, respectively, compared with the control (0.3 ± 0.08). Furthermore, p62 mRNA was increased by a factor of 0.92 ± 0.07 *p* ≤ 0.05, 1.7 ± 0.07 *p* ≤ 0.0001, 0.72 ± 0.08 *p* ≤ 0.5, and 2.1 ± 0.1 *p* ≤ 0.0001 compared with the control value of 0.72 ± 0.08 [46].

To transport proteins via the buccal mucosa, chitosan-maleimide (CSMHA)-coated liposomes were developed in 2022 by Sahatsapan’s group. The liposomes were preloaded with a protein analog, fluorescein isothiocyanate–albumin conjugate (FITC-BSA). Thin-film hydration was used to produce liposomes and CSMHA was used to adorn them. Liposomes coated with CSMHA were tested for cytotoxicity against oral fibroblast cells, biocompatibility, mucoadhesiveness on porcine buccal mucosa, loading efficiency and capacity, drug release, penetration via the buccal mucosa, protein integrity, and mucosal permeability. Liposome compositions with diameters between 35 and 166 nm were obtained. The maximum retention was observed for CSMHA-coated, FITC-BSA-loaded liposomes on buccal tissue. FITC-BSA penetration was highest when encapsulated in CSMHA-coated liposomes, which were non-toxic to healthy gingival fibroblast cells and buccal tissue. The results indicate that CSMHA-coated liposomes may be able to boost protein delivery via the buccal route [42].

### 3.3. Micelles

The encapsulation of hydrophobic drugs is a common application of polymeric micelles, which are nanosystems consisting of a hydrophobic core and a hydrophilic shell. The ability of these systems to protect the drug from the harsh GIT environment to increase drug stability renders them viable oral delivery systems. The high drug encapsulation capacity of polymeric micelles is also an advantage because a therapeutic dose can be attained rapidly while minimizing unwanted side effects and evading the epithelial efflux pumps. Because chitosan can be easily modified into an amphiphilic polymer with self-assembly abilities, it is widely used for the fabrication of polymeric micelles. Drug absorption is improved in the intestines because of chitosan’s mucoadhesive properties and its ability to temporarily open the tight connections of the epithelium [47].

The development of drug delivery systems using 3D printing technology has increased along with applications for individualized treatment. In nanomedicine, the capacity to design individualized structures loaded with drugs and delivery systems with a suitable drug dosage is of particular interest. To protect nanosystems from the harsh GIT environment, Almeida and colleagues (2021) coupled chitosan-based polymeric micelles loaded with camptothecin (CPT) into 3D printing systems (printfills) sealed with an enteric layer. The printfills were stable at a pH equivalent to that of simulated gastric fluid of the stomach and only released their micelles in the colon. The intestinal absorption was then recreated using the dissolving media and chitosan micelles significantly increased CPT permeability compared with the free drugs, with an apparent permeability coefficient (Papp) of approximately 9 × 10^−6^ cm/s in a 3D intestinal cell-based model. Colon-specific release of polymeric micelles using 3D printing and nanotechnology has the potential for enhancing intestinal absorption and preventing systemic or drug-specific degradation throughout the gastrointestinal tract [48].

Although chitosan is frequently used as a permeation enhancer for oral drug administration, it has some limitations, including an inability to form micelles and a lack of augmentation of drug bioavailability. In 2020, Tu et al. designed a novel chitosan derivative (gallic acid-Chitosan-D-α-tocopherol polyethylene glycol 1000 succinate (GA-CS-TPGS copolymer)) and constructed paclitaxel micelles (PTX-Micelles) with the goal of improving the bioavailability and anti-tumor efficacy of PTX (inhibition of P-gp efflux and drug metabolism in the liver) via the enhanced micelle solubility and permeability. Compared with Taxol^®^ (Bristol-Myers Squibb, Princeton, NJ, USA), PTX-Micelles were more effective against lung tumors and their bioavailability was increased by approximately 3.80-fold [49].

To improve the biopharmaceutical performance of drugs that are not highly water soluble, Kumar and colleagues synthesized a novel polymer consisting of oleic acid grafted to low molecular weight carboxymethyl chitosan (OA-CMCS). Analytical methods such as 1H-NMR and FT-IR spectroscopy were used for the design and synthesis of this polymer via an amidation reaction, and the resulting material was characterized. There was a low critical micellar concentration of 1 µg/mL for the OA-CMCS conjugate when dissolved in water. Docetaxel (DTX), a drug with low solubility in water, was selected as an appropriate example. Following parameter optimization, spherical DTX-loaded OA-CMCS micelles with a size distribution of 213.4 ± 9.6 nm and an entrapment efficiency of 57.26 ± 1.25% were obtained. An in vitro release evaluation was carried out using a body fluid model. DTX-loaded OA-CMCS micelles exhibited a gradual and prolonged DTX release behavior. Using an in vitro Caco-2 cell model, the ability of DTX-loaded OA-CMCS micelles to pass through the intestinal barrier was examined. Paracellular uptake of DTX was enhanced by as much as 6.57-fold when DTX was loaded into OA-CMCS micelles, as measured by their apparent permeability. Furthermore, an in vivo pharmacokinetic study showed that DTX-loaded OA-CMCS micelles exhibited a significantly higher Cmax (1.97) and AUC (2.62) compared with the free DTX suspension [50]. To enhance the oral absorption of doxorubicin (DOX), Yang and coworkers presented a new delivery technique in 2023 based on linolenic acid (LA)-linked chitosan (CS) polymeric micelles. To enclose DOX, the transporter ligand LA was grafted onto CS to create CS-LA micelles. The micelles were perfectly spherical and had a CMC (critical micelle concentration) of 23.6 μg/mL. The particle size of the micelles was 117.1 ± 1.6 nm, and the drug loading was 8.77 ± 0.02%. A relative bioavailability of 166% compared with DOX•HCl was observed in pharmacokinetic studies. The intracellular uptake, immunofluorescence imaging, and penetration test all confirmed the fatty acid transport protein 4-mediated intestinal absorption process of micelles. These results demonstrated that CS-LA micelles have the potential to function as an efficient oral delivery vehicle and fatty acid transporter is a promising target for improving intestinal drug absorption [51]. Micelles were synthesized by Zlotnikov et al. in 2023 using chitosan (or cyclodextrin) and oleic acid with varying degrees of modification. Micelles were analyzed via FTIR and fluorescence spectroscopy with a pyrene label (using a monomer-excimer method) to examine the incorporation of the antibacterial drugs moxifloxacin and rifampicin. When the antibiotics moxifloxacin and rifampicin are loaded into micelles, the fluorescence properties change. The maximum fluorescence emission moves to the long-wavelength region, whereas the fluorescence anisotropy increases because the hydrodynamic volume of the fluorophore-containing rotating fragment increases significantly. The pyrene label was used to calculate the critical micelle concentrations, which varied from 4 to 30 nM depending on the polymer selected. Micellar systems improve antibiotic efficacy by facilitating the drug’s uptake by bacteria and encasing it into a protective layer. Experiments on the pharmacokinetics of moxifloxacin in micellar systems demonstrated a 1.7-fold improvement in efficacy over the free form and a maximum concentration in the blood that was two-fold higher [52].

## 4. Applicability of Chitosan-Oriented Multifarious Delivery

With respect to oral non-viral gene delivery systems, halloysite nanotubes–carbon dots hybrids, chitosan–zein hybrid systems, chitosan-*p*-hydroxyphenacyl (CH-*p*HP), and poly(ethylene glycol)-poly(ε-caprolactone) copolymer (PEG-PCL) nanoparticles have been evaluated for effective oral delivery for many pathological conditions, including inflammatory bowel disease, diabetes mellitus, obesity, and post-traumatic osteoarthritis [53,54]. Among the various types of nucleic acids, DNA, siRNA, miRNA, and oligonucleotides can be incorporated to develop facile and low-cost systems compared with other routes of administration [55]. For example, Jabali et al. [56] invented a low-cost plasmid DNA (pDNA)-based oral nanoparticulate system utilizing ascorbic acid-derivatized, chitosan-coated superparamagnetic iron oxide nanoparticles (SPION) for vaccine delivery. They observed that pDNA release was favorable and reached 45% after 48 h. Thus, chitosan can successfully encapsulate pDNA in the extremely acidic stomach environment but allows release while passing through the target alkaline intestinal cells. 

In another study, Wang et al. [57] evaluated chitosan-based tripolyphosphate (CS-TPP) nanoparticles for combinatorial therapy of methylenetetrahydrofolate dehydrogenase 1-like shRNA (Short hairpin RNA)(MTHFD1L) and 5-aminolevulinic acid (ALA) for dual photodynamic gene transfer in oral cancer. The effect of concomitant delivery of shRNA/photosensitizer on gene expression in oral squamous cell carcinoma cells was assessed and resulted in apoptosis and the generation of reactive oxygen species.

Structurally modified chitosan after methylation or thiolation significantly improved mucoadhesion and penetration behavior because of its ability to open tight junctions between epithelial cells and efficiently deliver proteins and peptides [58]. Although intrinsically weak in structure, with increased molecular weight, and more hydrophilicity, these macromolecules are administered parenterally or subcutaneously. Currently, studies are being conducted to deliver them orally [38]. Many studies have been carried out to evaluate the delivery of peptides and proteins, including thyrotropin-releasing hormone, octreotide, desmopressin, vasopressin, uroguanferin, calcitonin, exenatide, human insulin, parathyroid hormone, leptin, interferon, and ovalbumin using different polymers [59]. Various chitosan-based delivery systems, such as microparticles, nanoparticles, liposomes, and niosomes have been developed to deliver proteins [60,61,62]. In fact, chitosan is actively studied as a vehicle for oral insulin drug delivery, which is economical and more suitable to patients compared with injectables [63]. Electrostatic attractions between positively charged chitosan and negatively charged insulin induce the formation of self-assembled nanoparticles. This system helps to protect the core insulin molecules from enzymatic deterioration within the stomach and promotes paracellular intestinal uptake from enterocytes because of mucoadhesiveness and reversible tight junction opening [64]. Furthermore, modified chitosan nanoparticles have resulted in novel methods for improved targeting and sustained delivery [65]. The oromucosal approach is appealing since it is minimally invasive. Salivary flow and the mucosa provide a major permeability barrier that inhibits drug absorption. In this direction, Stie and his team in 2023 developed a multi-layered nanofiber-on-foam-on-film (NFF) drug delivery system with unique properties based on polysaccharides combined as (i) mucoadhesive chitosan-based nanofibers, (ii) a peptide loaded hydroxypropyl methylcellulose foam, and (iii) a saliva-repelling backing film based on ethylcellulose. The dynamic mechanical study showed that NFF has superior mechanical qualities, and exposure to a monolayer of TR146 cells reveals its biocompatibility. The mucoadhesion of the NFF was enhanced by the addition of chitosan-based nanofibers. After 1 h, the NFF released more than 80% of the peptide desmopressin. Desmopressin penetration was enhanced by NFF compared to a commercial freeze-dried tablet in ex vivo permeation trials across porcine buccal mucosa. Evidence from this study supports further investigation into the NFF’s viability as a biocompatible drug delivery system [66].

**Figure 2:**
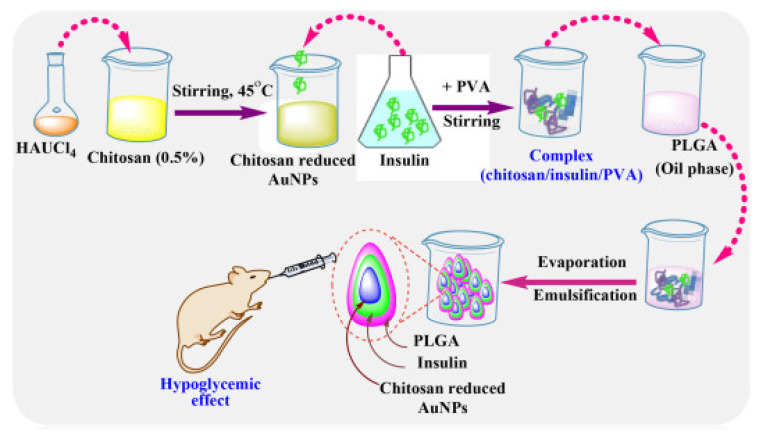
Diagrammatic representation of the in situ synthesis of gold nanoparticles (NPs) onto chitosan-modified PLGA nanoparticles for the oral delivery of insulin. (Reprinted from [67], with permission from Elsevier).

Interestingly, Asal et al. [67] conducted a comparison study between three different biocompatible nanoparticulate systems, including pure chitosan, chitosan with gold, and chitosan gold-modified poly lactic-*co*-glycolic acid (PLGA) for the fabrication of oral insulin (Figure 2). Insulin was retained for approximately 96% at stomach pH in the case of PLGA-modified nanoparticles and subsequently exhibited intestinal sustained release. The hypoglycemic effect was noted by calculating blood glucose levels decreasing to 38% for pure chitosan NPs, 35% for chitosan with gold NPs, and 27% for nanoparticles with chitosan-modified PLGA in animal studies.

**Figure 3:**
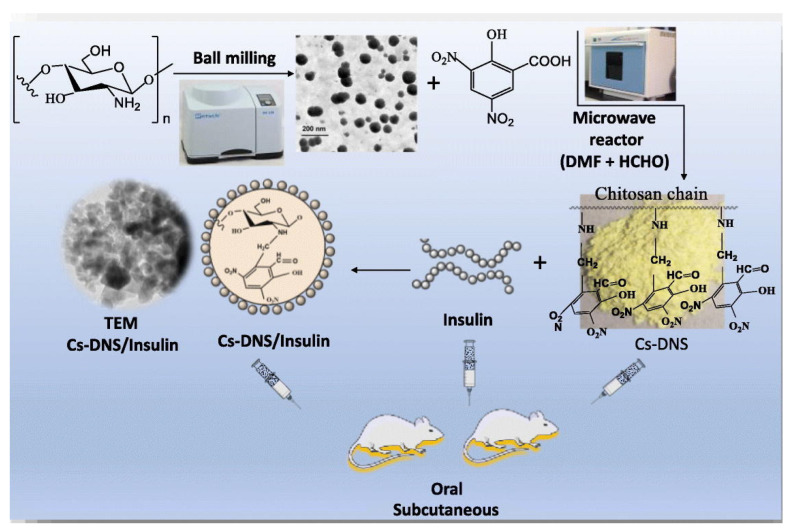
Scheme depicting the synthesis of reduced chitosan (Cs) modified with dinitrosalicylic acid (DNS) by microwave irradiation method for oral insulin. (Reprinted from [68], with permission from Elsevier).

In another study, the chitosan was initially reduced to a smaller particle size and later modified in the presence of dinitrosalicylic acid to increase the oral insulin potency in the presence of microwave irradiation (Figure 3). The functionalized nanocarriers exhibited a hydrodynamic diameter of approximately 33 nm and +35 mV of zeta potential, regardless of entrapped insulin. Moreover, 92% of insulin retained at a lower pH inside the nanocarrier was subsequently released at higher pH values. Reduced blood glucose levels of nearly 39% and a bioavailability of 17% were observed over a period of 3 h using the synthesized system [68].

**Figure 4:**
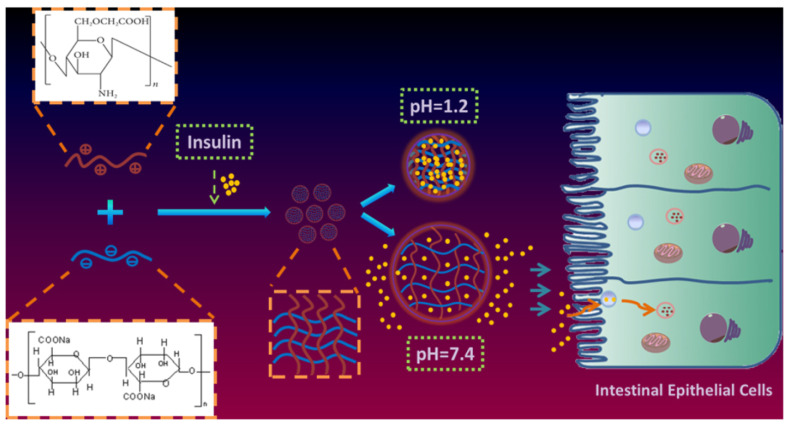
Image showing a pH-modified nanohydrogel (*O*-carboxymethyl chitosan/sodium alginate) for augmented oral insulin delivery. (Reprinted from [69], with permission from Elsevier).

Zhang et al. [69] constructed *O*-carboxymethyl chitosan in the presence of sodium alginate (SA)-based nanohydrogel (Figure 4). This system also exhibited a similar pH release profile for insulin and encouraging results in type 1 diabetic animal studies with a pharmacologic bioavailability of 6.57%.

### Role of P-gp Inhibitors in Oral Drug Delivery

Permeation-glycoprotein (P-gp) inhibitors play a crucial role in enhancing the oral delivery of drugs formulated in nanoparticle systems [70]. P-gp is a membrane transporter protein found in the gastrointestinal tract that actively pumps drugs out of the enterocytes, thereby limiting their absorption and bioavailability. Nanoformulations such as polymeric nanoparticles and lipid-based carriers gained significant attention as oral drug delivery systems due to their ability to improve drug solubility, stability, and target-specific delivery [71,72]. P-gp inhibitors, when co-administered with nanoformulated drugs, inhibit the efflux activity of P-gp, allowing for increased drug absorption and improved therapeutic outcomes. By blocking P-gp, these inhibitors prevent the rapid elimination of drugs from the gut and enhance their residence time in the absorption site, thereby increasing their absorption efficiency [73]. Furthermore, P-gp inhibitors can also modulate the tight junctions between enterocytes, promoting paracellular transport and facilitating drug permeability. This mechanism further contributes to the improved oral bioavailability of nanoformulated drugs [74]. By increasing drug absorption, prolonging residence time, and improving bioavailability, P-gp inhibitors serve an integral part in nanoformulation oral drug delivery. Incorporating them into nanoparticle-based systems has the potential to significantly improve therapeutic efficacy and overcome the drawbacks of oral drug administration [75].

Therapeutic approaches that target other factors in modified biochemical or pharmacological pathways are among the methods utilized to overcome drug resistance. P-glycoprotein (P-gp) and multidrug resistance proteins are two of the numerous factors that contribute to the development of drug resistance. Formulations, pharmaceutical excipients, and tailored compounds are all examples of P-gp inhibitors. As a result of their beneficial effect on P-gp, functionalized polymers such as chitosan, soluplus^®^, poloxamers, polyethylene glycols, dendrimers, anionic gums, sodium alginate, and others could be used as drug delivery tools [76]. Due to the importance of P-glycoprotein (P-gp) in the active transport of different substrates with different structures outside of cells, intestinal permeability is reduced, and bioavailability is decreased after oral administration. Many P-gp substrates have had their oral absorption and bioavailability improved via the application of P-gp inhibitors, which have varied from small molecule drugs and natural ingredients to pharmaceutically inert excipients. Drug–drug interactions and enhanced adverse effects are possible when P-gp substrates and small molecule P-gp inhibitors are given together. On the other hand, inert excipients including polymers, surfactants, and lipid-based excipients are entirely innocuous, pharmaceutically acceptable, and are not absorbed by the body. Notably, the formulation itself and the P-gp inhibitory effects of the excipients can work together to improve drug solubility, absorption, and bioavailability when used in pharmaceutical formulations. Several other formulations have been developed that already contain P-gp inhibitory action. Some examples are micelles, emulsions, liposomes, polymeric nanoparticles, microspheres, dendrimers, and solid dispersions. Different processes based on their characteristics allow them to avoid P-gp [77]. Wande et al., in 2022, reported a nanogel-polymersomes drug delivery system based on modified chitosan, chitosan diacetate (CDA), methoxy-poly-(ethylene glycol)-b-poly (lactide) (MPP), and D-alpha-tocopheryl polyethylene glycol succinate (TPGS) with permeation-glycoprotein (P-gp) inhibition capability for enhancing the oral chemotherapy efficacy of erroneously soluble rapamycin and its synergistic chemotherapy with Oxaliplatin. The nanogel-polymersomes drug delivery system has tremendous potential for future cancer chemotherapy due to its surpassed Oxaliplatin, rapamycin, and Oxaliplatin and rapamycin mixtures (OR) in vitro cytotoxicity and in vivo antitumor chemotherapeutic efficacy [78]. In 2019, Mu and his team synthesized quercetin–chitosan conjugate (QT-CS) for oral delivery of doxorubicin (DOX) to improve its oral bioavailability by increasing its water solubility, opening tight junction and bypassing the P-glycoprotein (P-gp) [79]. In 2023, Nielsen and his team proposed that etoposide and zosuquidar could work together in the small intestine lumen by simultaneous co-release and subsequent spatiotemporal linkage. To this end, freeze-dried etoposide and zosuquidar aqueous suspension dosage forms (ASDs) have been synthesized in polyvinylpyrrolidone (PVP), hydroxypropylmethyl cellulose (HPMC) 5, and HPMC 4 k. Overall, the research showed that a unique and effective formulation technique to boost the bioavailability of P-gp substrates may be the simultaneous co-release of an amorphous P-gp substrate and inhibitor [80].

## 5. Herbal Bioactives Loaded Nanoformulations for Oral Delivery

**Figure 5:**
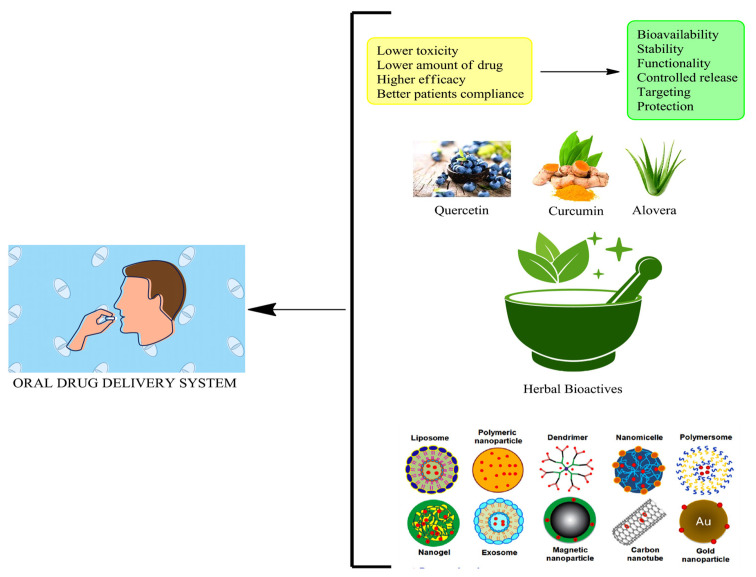
Illustration depicting the application of herbal bioactive nanoformulations in oral drug delivery systems. The nanoformulations enable enhanced solubility, stability, and targeted delivery of herbal bioactive compounds, improving their therapeutic efficacy.

Nearly 80% of the world depends on traditional herbal medicines for healthcare purposes because they may be used to treat a variety of ailments [81]. According to the World Health Organization, approximately 25% of modern medicines are derived from plants [82]. Herbal bioactive drug delivery systems have emerged as a promising approach to oral drug delivery. These systems leverage the therapeutic potential of natural compounds derived from herbs, which exhibit diverse biological activities [83]. By encapsulating these bioactive compounds within delivery systems such as nanoparticles, liposomes, or micelles, their stability, bioavailability, and targeted delivery can be improved. The use of herbal bioactive drug delivery systems offers several advantages, including enhanced solubility, sustained release, reduced toxicity, and improved therapeutic efficacy. Furthermore, these systems can be tailored to specific herbal extracts, allowing for personalized medicine approaches [84,85]. Continued research in this field holds great potential for developing safe and effective oral drug delivery systems (Figure 5).

### 5.1. Aloe Vera

Aloe vera (AV) belongs to the Liliaceae family, of which the best-known species is Aloe Barbadensis Miller [86]. Aloe vera extract helps to improve oral bioavailability and acts as a good bio-enhancer [87]. Also, AV has the potential to treat diseases, such as metabolic, cardiovascular, tumor, and oral diseases, and has other applications as shown in Table 1 [88].

Patri et al. compared and evaluated herbal agents for intercanal antimicrobial activity. For this study, 80 extracted human premolar teeth were decoronated to obtain 6 mm blocks of the mid root. The samples were treated for 21 days with *E. faecalis*, which may be divided into Group 1 (control, Triple antibiotic paste), Group 2 (curcumin), Group 3 (Propolis), and Group 4 (Aloe vera gel), which were further categorized as chitosan present or absent. One-way ANOVA and Tukey’s Post hoc test revealed that propolis exhibited antimicrobial properties with similar efficacy (*p* = 0.598), while formulation triple antibiotic paste (TAP) containing chitosan–curcumin conjugate showed (*p* = 0.963), and aloe vera gave the poorest result, but on the addition of chitosan, there was a significant increase (*p* = 0.000) in the antimicrobial efficacy. As AV yielded poor results when mixed with chitosan, the antimicrobial efficacy was enhanced; thus, it acted as a promising agent for drug delivery [89].

Khan et al. [90] prepared a hydrogel based on ofloxacin (OX) at concentrations of 0.5, 2.5, and 5 mg loaded into chitosan (CS) and AV using a freeze gelation method. The results indicated that 0.5 mg OX hydrogel showed enhanced proliferation of 2T3 fibroblast cells, sustained drug release, and antibacterial activity compared with 2.5 and 5 mg OX hydrogels. All hydrogels exhibited anti-angiogenic effects, but higher activity was exhibited at lower concentrations (i.e., 0.5 mg OX hydrogel). Therefore, hydrogels represent a promising wound dressing for future purposes.

Ajaz et al. [91] developed a pH-responsive, semi-interpenetrating polymer network (semi-IPN) of itaconic acid-grafted polyacrylamide plus aloe vera gel [IA-g-poly-(Aam)/aloe vera] for the delivery of cetirizine HCl (CTZ HCl) to the lower GIT. The F5 formulation consisting of 0.3% AV and 6% IA was selected based on sol-gel and swelling analysis. An in vitro evaluation revealed the release of F5 in acidic media and controlled release in the intestine within 72 h. Thus, because of the non-toxic nature of the formulation, it has potential as a targeted drug carrier.

**Table 1:** Aloe vera and chitosan-blended nanoformulations and their applications.

Components	Nanoformulations	Applications	Reference
Aloe vera + Chitosan + polycaprolactone	Nanofiber	Antibacterial	[92]
*Aloe marlothii* gel + Chitosan	Nanoparticles	Antioxidant, Anti-inflammatory, anti-apoptotic	[93]
Chitosan + *Aloe vera*	Silver Nanoparticles	Enhanced physiochemical properties	[94]
Aloe vera + Chitosan + Dextran sulfate + Eucalyptus extract	Nano-hydrogel	Antibacterial	[95]
Aloe vera + Chitosan + Minoxidil	Nanocomposite	Alopecia therapy	[96]

### 5.2. Quercetin

Quercetin (QC) is a flavonoid derived from plants and is present in fruits and vegetables in high amounts [97]. One of the most important medicinal properties of QC is its anticancer activity [98].

Sun et al. prepared Quercetin-loaded chitosan and sodium alginate nanoparticles (Q-CSNPs) using a single factor experiment method. Antibacterial activity was observed at five different nanoparticle concentrations against *E. coli* and *S. aureus*. α, α-diphenyl-β-picrylhydrazyl (DPPH) hydroxyl radical scavenging experiments showed antioxidant activity, and the NPs inhibited oxidative stress resulting from lipopolysaccharide (LPS) exposure. The results indicated that Q-CSNPS exhibited good antibacterial and antioxidant activity. Therefore, this preparation warrants further evaluation, and other quercetin nano-drugs are under development as shown in Table 2 [99].

Essa et al. [100] prepared Quercetin-loaded Poly Lactic-co-Glycolic Acid (PLGA) nanoparticles coated with chitosan to enhance Prostate-specific membrane antigen (PSMA)-specific activity against Lymph Node Carcinoma of the Prostate (LNCap) prostate tumor cells. An in vitro release study revealed that this nano-system provided pH-dependent, cellular uptake as well as sustained and higher cytotoxicity compared with the non-targeted nano-system. The results suggest that this nano-system is an efficient nanocarrier for targeted drug delivery and quercetin release.

Bhadraiah et al. examined albendazole (ABZ) pharmacokinetics, which was administered orally with quercetin (QUE) in chitosan-alginate (CS-ALG) encapsulated microspheres to broiler chickens. Approximately 32 broilers were divided into the following four groups (G): G1 treated with 10 mg/kg ABZ orally, G2 with ABZ intravenously, G3 with CS-ALG-ABZ, and G4 with CS-ALG-ABZ-QUE. Group IV exhibited a significantly high area under the curve (AUC) and mean residence time (MRT) (~2.5 fold) compared with that of the other groups. Therefore, the results indicated that QUE enhanced ABZ absorption and reduced metabolite formation by inhibiting intestinal/hepatic cytochrome (CYPs). The results showed a prolonged absorption and increase of maximum concentration (Cmax), Mean residence time (MRT), and AUC in the quercetin-containing formulation, suggesting that it represents a promising formulation for future use [101].

**Table 2:** Chitosan and quercetin nanoformulations and their applications.

Components	Nanoformulations	Applications	Reference
Chitosan + Quercetin (QC) + Alginate + Zein	Nanoparticles	Delivery system	[102]
QC + Chitosan + Doxorubicin (DOX)	Nanoparticle	Cardiotoxicity	[103]
Quercetin + CS + Aspirin + Eudragit L100	Nanoparticles (NPs)	Colorectal cancer	[104]
Quercetin + Chitosan	NPs	Anti-rheumatic	[105]
Quercetin + Chitosan + Halloysite (HNT) + Graphite-carbon nitride (g-C_3_N_4_)	Hydrogel nanocomposite	Anticancer	[106]

### 5.3. Curcumin

Curcumin (CUR) is a polyphenolic molecule derived from the rhizomes of *Curcuma longa* [107]. It has been widely studied as a potential treatment for cancer, fungi, viruses, arthritis, bacterial infection, allergies, Alzheimer’s disease, and other chronic disorders as listed in Table 3 [108,109]. It also exhibits anti-inflammatory and antioxidant activity.

Sourour et al. [110] prepared curcumin and succinylated curcumin (CUR.SA) encapsulated in mannosylated chitosan nanoparticles (CMNPs) to form CUR-NPs and CUR.SANPs, respectively, and assessed their activity against colon cancer. Both NPs exhibited dose and time-dependent cytotoxicity toward HCT116 and SW480 cell lines. Western blot analyses revealed apoptosis induction via caspase signaling activation compared with untreated cells. Further in vitro and physiochemical studies revealed that the NP formulations represent a potential treatment for cancer.

Abduh et al. [111] analyzed nanoformulations consisting of CD44-targeted cyclosporine (CsA)-loaded thiolated chitosan (TC) coated with hyaluronic acid (HA) for sustained release in triple-negative breast cancer (TNBC). After interacting with the polymer, the particle size and zeta potential were 192 nm (0.433 PDI) and 38.9 mV, respectively. The formulation exhibited approximately 85% encapsulation of drug and drug dissolution at a low pH (7.4 and 6.8) and exhibited significant cytotoxicity at lower concentrations against TNBC and normal breast epithelial cells. Based on these characteristics, this NF represents an effective and potent treatment for cancer.

Dash et al. [112] synthesized chitosan-modified polylactic-co-glycolic acid (PLGA) atorvastatin–curcumin conjugate (AT-CU) nanoparticles for the management of atherosclerosis. Upon increasing the concentration of chitosan-modified PLGA AT-CU NPs, the particle size increased from 139.2 nm to 197.7 nm, the zeta potential ranged between 20.57 mV and 28.32 mV, and the drug encapsulation efficiency was enhanced from 71.81 to 90.57%. After 18 h, the burst release of the NPs reached 70.8%. Thus, further study of this formulation for the diagnosis of atherosclerosis is warranted.

**Table 3:** Chitosan–Curcumin-blended formulations.

Components	Nanoformulations	Applications	Reference
Curcumin + Chitosan + Rose Bengal (RB)	Niosomes	Lung cancer	[113]
Chitosan + Curcumin + Cerium oxide + Octenyl-succinic anhydride (OSA)	Nanoparticles	Antibacterial, anti-inflammatory	[114]
Chitosan + Hyaluronic acid + Curcumin + Cisplatin	Nanoparticle	Cervical carcinoma	[115]
Chitosan + Curcumin + Trimethoprim-sulfamethoxazole (TMP-SXT)	Nanocomplexes	Antimicrobial, Antibiofilm	[116]

## 6. Patents

There is significant potential for expansion in this area of research, particularly when one considers the limitations of the various available techniques and systems. Table 4 lists the patent applications associated with different biopolymeric nanoformulations used for oral drug delivery. Ali et al., in 2022, claimed that Nanoparticles (NPs) coated with alpha-1 acid glycoprotein (AGP), an anti-inflammatory protein, have been shown to inhibit tumor metastasis and invasion while also circumventing breast cancer cells’ resistance to treatment. The sequential ionic gelation, spray-drying, and AGP-surface adsorption processes for producing hyaluronic acid-chitosan nanoparticles decorated with AGP (AGP-HA NPs) are also reported. The survival rate of patients with metastatic breast cancer may be increased by employing the disclosed AGP-HA NPs containing drugs in combination with standard chemotherapy [117]. Yan et al., in 2022, disclosed the method of developing a high-load oral paclitaxel capsule for a slow release in the colon, which comes under a broad category of porous starch drug loading [118]. Dong and inventors, in 2020, claimed that the present invention pertains to an eye drop and a method for making the same that contains chitosan nanoparticles encapsulating polydeoxyribonucleotide (PDRN). This invention focuses on mucoadhesive nanoparticles and a technique for making them that can increase the drug’s residence time in the ocular mucous membrane, where it can be more effectively delivered [119]. Porous structure-based magnetically actuated microrobot, as well as a method for fabricating the same, wherein the porous structure-based magnetically actuated microrobot is based on a biocompatible and biodegradable natural polymer, allowing for precise targeting of the porous microrobot via the attachment of magnetic nanoparticles, as well as the delivery of drugs and cells using the microrobot [120]. Shadab and his team in 2021, claimed nanoparticles of soy protein and a bioactive ingredient are encapsulated in a sodium alginate film to form an in situ gelling composition. If a patient requires a prolonged release of the bioactive agent, the in situ gelling composition can either be added to a solid dosage form or reconstituted with water for oral administration [121]. In accordance with the disclosure, a nanoparticle comprising an alginate-oleic acid particle is provided that encapsulates and delivers two different antiviral drugs, one of which is remdesivir. The therapeutic application of the nanoparticle to the treatment of viral diseases such as SARS-CoV-2 is also discussed in the disclosure [122]. For the purpose of delivering nucleic acids, such as gene transfer, in vivo, the present invention reported by Jun in 2019 generally pertains to nanoparticles including dually derivatized chitosan, and methods of manufacturing and employing the same [123]. The field of composition for cancer care is broadly related to this investigation. Specifically, an oral cisplatin nanoparticle formulation is the focus of the innovation. A new method of making the compound was also part of the invention [124]. Nanocapsules with a chitosan coating, and a method of using them, are the subject of this invention. An effective method for preparing nanocapsules with a particle size of 500 nm or less is described, and nanocapsules containing a poorly soluble drug are loaded using the method, resulting in a high rate of skin permeability for the drug-containing nanocapsules, drug delivery into the skin, and drug efficacy. Nanocapsules coated with chitosan of the present invention are expected to be used to develop an excellent delivery system, of which the delivery efficiency of a poorly soluble drug or the active ingredients to animals, and companion animals, is significantly increased, in the pharmaceutical field, the cosmetics industry, the food industry, and the field of veterinary science [125]. In 2020, Yuriy and Vladislav claimed the new cross-linked chitosan, preparations, formulations, and uses thereof. Nanoparticles, and compositions containing them, are of particular interest because of their potential as active agents and delivery systems for several biologically active substances [126].

## 7. Future Perspectives

Recent advancements in chitosan nanoformulations for oral drug delivery systems have opened up promising possibilities for future directions in pharmaceutical research. Chitosan, a natural polymer derived from chitin, possesses excellent biocompatibility, biodegradability, and mucoadhesive properties, making it an ideal candidate for enhancing drug absorption and bioavailability. Moving forward, researchers can focus on optimizing chitosan nanoformulations to overcome the limitations of conventional oral drug delivery systems. This includes exploring strategies to improve drug loading efficiency, stability, and controlled release kinetics. Furthermore, the development of multifunctional chitosan nanoparticles that can target specific sites within the gastrointestinal tract or deliver multiple drugs simultaneously holds great potential. Additionally, efforts can be directed toward enhancing the understanding of the interaction between chitosan nanoparticles and biological barriers in the gastrointestinal tract. This would involve investigating factors such as mucus penetration, cellular uptake mechanisms, and potential toxicity concerns. Moreover, combining chitosan nanoformulations with other advanced technologies such as nanotechnology, biotechnology, and personalized medicine can further revolutionize oral drug delivery systems. This may involve incorporating stimuli-responsive or smart systems to achieve site-specific drug release, utilizing nanoscale carriers for co-delivery of drugs and imaging agents, or integrating chitosan nanoparticles with targeted therapeutic approaches. In a nutshell, future directions in chitosan nanoformulations for oral drug delivery systems involve continuous optimization, the understanding of biological interactions, and integration with other cutting-edge technologies. These advancements have the potential to revolutionize drug delivery, improve patient compliance, and enhance therapeutic outcomes in the field of pharmaceutical research.

## 8. Conclusions

Despite the fact that drugs must pass through multiple barriers and circumvent biological processes that decrease their bioavailability and efficacy, oral administration remains the preferred drug delivery method for both patients and physicians. Nanoformulations or nanoparticulate drug delivery techniques using biocompatible polymers have gained significant interest. Their physicochemical properties make them suitable for use as nontraditional methods of dosing. In addition, they enable vector processing of drug molecules with low solubility and bioavailability, thus enhancing their interaction with the target organs or cells via alternative routes of administration. Unconventional treatment typically utilizes chitosan-based NPs because of their low cost, high efficiency, and ability to encapsulate peptides, therapeutics, and DNA to disrupt certain biological processes. This can be accomplished orally without the need for invasive or painful routes of administration because chitosan NPs can overcome physical and biological barriers to increase drug bioavailability, resulting in higher efficacy with fewer adverse reactions.

## Figures and Tables

**Table 4:** List of published patents on Chitosan-based nanosystems for oral drug delivery.

S.No.	Patent Number	Title	Inventors	Year	Reference
1	US20230248659A1	Immune-suppressing nanoparticles for robust sensitization of drug-resistant cancer	Ali H. ALHASAN Haneen OMAR, Roa’ FARDOUS, Yasser ALHINDI, Abdulaziz Almalik, Waleed M. ALGHAMDI	2022	[117]
2	US20230233511A1	Method for Preparing High-load Oral Paclitaxel Capsule for Slow Release in Colon	Yan Hong, Beibei Zhao, Zhengbiao Gu, Li Cheng, Zhaofeng Li, Caiming Li, Xiaofeng BAN	2022	[118]
3	US20220160624A1	Eye drops containing chitosan nanoparticles in which pdrn is encapsulated, and the preparation method therefore	Dong Rack Choi, De Zoysa Pathmendrra Mahanama, Thi Thu Thao Nguyen, Ji Soo Lee, Sajith Dananjaya Sirimanna Hettilage	2020	[119]
4	US20220305243A1	Chitosan porous structure-based magnetically actuated microrobot	Jong Oh Park, Chang Sei KIM, Eun Pyo CHOI, Gwang Jun GO, Hyeong Woo SONG, Yeong Jun CHANG, Ami Yoo	2020	[120]
5	US11260025B1	In situ gelling composition as a pH-selective and mucoadhesive sustained release drug delivery system	Shadab Md, Samaa T. Abdullah, Nabil A. Alhakamy	2021	[121]
6	US20230233473A1	Synergistic anti-viral pharmaceutical composition containing targeting nanoparticles	Chung Chin SUN, Dean Mo Liu	2021	[122]
7	US11623011B2	Dually derivatized chitosan nanoparticles and methods of making and using the same for gene transfer in vivo	Jun Gao, Eric Hsu, Anthony Cheung	2019	[123]
8	US20230092662A1	Cisplatin nanoparticle composition, method for the preparation thereof	Sarasija Suresh, Vishal Uchila Shishir Rao, S. Narasimha Murthy, H.N. Shivakumar	2018	[124]
9	US20220054425A1	Nanocapsules coated with chitosan and use thereof	Won Il Choi, Sung Hyun Kim, Yong Chul Shin, Jeung Hoon Lee, Jin Hwa Kim, Young Sung Yun	2019	[125]
10	US20230096466A1	New method of synthesis of chitosan derivatives and uses thereof	Yuriy Kargapolov, Vladislav FOMENKO	2020	[126]
